# Repetitive Transcranial Magnetic Stimulation With H-Coil in Alzheimer's Disease: A Double-Blind, Placebo-Controlled Pilot Study

**DOI:** 10.3389/fneur.2020.614351

**Published:** 2021-02-18

**Authors:** Letizia Leocani, Gloria Dalla Costa, Elisabetta Coppi, Roberto Santangelo, Marco Pisa, Laura Ferrari, Maria Paola Bernasconi, Monica Falautano, Abraham Zangen, Giuseppe Magnani, Giancarlo Comi

**Affiliations:** ^1^Experimental Neurophysiology Unit, Institute of Experimental Neurology - INSPE, Hospital San Raffaele, Milan, Italy; ^2^University Vita-Salute San Raffaele, Milan, Italy; ^3^Neuropsychology and Clinical Psychology Service, Hospital San Raffaele, Milan, Italy; ^4^Neuroscience Laboratory, Ben-Gurion University of the Negev, Be'er Sheva, Israel; ^5^Memory Disorders Unit, Institute of Experimental Neurology-INSPE, Hospital San Raffaele, Milan, Italy; ^6^Institute of Experimental Neurology-INSPE, Hospital San Raffaele, Milan, Italy

**Keywords:** repetitive transcranial magnetic stimulation, Alzheimer's disease, ADAS-cog, H-coil, neuromodualtion

## Abstract

Focal repetitive transcranial magnetic stimulation (rTMS) has been applied to improve cognition in Alzheimer's disease (AD) with conflicting results. We applied rTMS in AD in a pilot placebo-controlled study using the H2-coil. H-coils are suitable for targeting wider neuronal structures compared with standard focal coils, in particular the H2-coil stimulates simultaneously the frontal-parietal-temporal lobes bilaterally. Thirty patients (mean age 70.9 year, SD 8.1; mean MMSE score 16.9, SD 5.5) were randomized to sham or real 10 Hz rTMS stimulation with the H2-coil. Each patient underwent 3 sessions/week for 4 weeks, followed by 4 weeks with maintenance treatment (1 session/week). Primary outcome was improvement of ADAS-cog at 4 and 8 weeks compared with baseline. A trend toward an improved ADAS-cog score over time was observed for patients undergoing real rTMS, with actively treated patients experiencing a mean decrease of −1.01 points at the ADAS-Cog scale score per time point (95% CIs −0.02 to −3.13, *p* < 0.04). This trend was no longer evident 2 months after the end of treatment. Real rTMS showed no significant effect on MMSE and BDI changes over time. These preliminary findings suggest that rTMS with H-coil is feasible and safe in patients with probable AD and might provide beneficial, even though transient, effects on cognition. This study prompts larger studies in the early stages of AD, combining rTMS and cognitive rehabilitation.

**Clinical Trial Registration:** www.ClinicalTrials.gov, identifier: NCT04562506.

## Introduction

Alzheimer's disease (AD) is a progressive neurodegenerative disorder affecting memory, language and several other cognitive functions, representing the leading cause of dementia. At present, only a few therapies are available for AD patients. Converging evidence suggests that neuromodulation with repetitive transcranial magnetic stimulation (rTMS) may be useful as an additional, non-invasive and safe treatment for AD (1, 2). Previous rTMS studies on AD were conducted with focal traditional figure-of-eight coils (3–11). High rTMS frequencies (>5 Hz), considered to have an excitatory effect (12) probably through synaptic long-term potentiation (LTP) mechanisms. Conversely, low frequencies (≤1 Hz) are associated with inhibitory effects and may act on cortical hyperexcitability (13, 14). One of the main brain target associated with cognitive improvement after excitatory rTMS in AD is the dorso-lateral prefrontal cortex (DLPFC) (3–5, 8, 15). Excitatory stimulation of other fronto-parietal targets has shown conflicting results (2, 16). Several functional neuroimaging studies showed indeed hyper-activation of the right DLPFC in subjects affected by AD and mild cognitive impairment (17–19), interpreted as a compensatory mechanism. Accordingly, high-frequency non-invasive brain stimulation over left and right DLPFC, applied either unilaterally (4, 20, 21) or bilaterally in sequence (3, 5, 8, 15), as well as low frequency stimulation of the right DLPFC have been reported to improve cognitive function in AD (16). Compared with focal coils, the Hesed coil (H-coil) is designed to reach wider brain regions, owing to a lower decay of the electric field with distance. Moreover, the H-coil allows simultaneous stimulation of different brain regions (22, 23). High-frequency rTMS with H-coil has been already applied in major depressive disorder (24), Parkinson's disease (25), language disorders (26–28), and stroke-related upper and lower limb motor deficits (29, 30). However, little is known about the potential usefulness of H-coil in AD. The H2-coil simultaneously stimulates medial pre-frontal, lateral frontal regions and temporal-parietal areas (31). The H2-coil rTMS has been reported promising in a small open-label case series (32). Our aim was to explore feasibility, safety and efficacy of excitatory rTMS of bilateral fronto-temporo-parietal regions with H2-coil in AD in a pilot randomized, placebo-controlled, double-blind study.

## Materials and Methods

### Participants

We enrolled thirty subjects referred to the Memory Clinic of our Institute for a diagnosis of probable AD according to NINCDS-ADRDA criteria (33). Since some patients were enrolled before 2011, all the diagnoses were reviewed according to the recent revision of the NINCDS-ADRDA criteria (34) at the end of the study. A record was judged to fulfill NINCDS-ADRDA criteria for probable AD if (a) the MMSE score was ≤ 24 and (b) there were demonstrable deficits in memory and at least one other area of cognition as defined by the criteria statement. Other inclusion criteria were: presence of a reliable caregiver and ability to sign a written informed consent. Exclusion criteria were: other neurological or psychiatric disorders accounting for the cognitive deficits; contraindications to TMS (35); therapeutic changes in the last 5 weeks; participation in other clinical trials in the previous 3 months. Patients fulfilling these criteria have been consecutively enrolled in the study. Two patients randomized to sham rTMS have been discarded from the study for a subsequent confirmed diagnosis of SCA17 and acute myocardial infarction prior to treatment start, respectively. All but two subjects were under conventional AD treatment at the therapeutic dosage: rivastigmine, (*n* = 7); donepezil, (*n* = 9) memantine (*n* = 2), rivastigmine and memantine (*n* = 7), donepezil and memantine (*n* = 2). Two subjects were not assuming any conventional anti-AD drugs for intolerance to the active molecule. All subjects and caregivers gave their written informed consent to participate in the study, which was approved by our Institutional Review Board.

### Study Design and H-Coil rTMS

The study was a double-blind, placebo-controlled paradigm. We used a 1:1 unrestricted randomization protocol to allocate participants to a real rTMS group or a sham (placebo) rTMS group (clinicaltrials.gov ID NCT04562506). Repetitive TMS was applied using the H2-coil (Brainsway Ltd., Jerusalem, Israel) designed to simultaneously target the bilateral frontal-parietal-temporal regions (31) coupled to a Magstim Rapid2 stimulator (Magstim Company Ltd., Whitland, Dyfed, UK). Sham stimulation was delivered through an alternative circuit through another coil in the same stimulation device, non-tangentially oriented to the scalp and with elements producing significant field cancellation. The sham coil induces an electric field <30% compared with the active coil but with similar acoustic artifact and scalp sensations, without effectively reaching the brain (36). The intensity of rTMS was chosen according to individual resting motor thresholds (RMT), defined as the minimal intensity able to evoke a reproducible motor-evoked potential (>50 μV) on the right abductor pollicis brevis, or any visible right hand movement, in at least 5 out of 10 stimuli. After having identified the hotspot over the left motor cortex and measured the RMT by tilting the coil in the medio-lateral direction, the coil was centered medially and moved 6 cm anteriorly along the nasion-inion line, in order to cover the bilateral prefrontal areas (25, 27) with right-left symmetry. At this position, the H2-coil ensures bilateral and simultaneous stimulation of structures both in the right and left prefrontal cortices and in temporal-parietal areas (31). For each rTMS session, 840 stimuli were delivered at 10 Hz (42 trains of 20 stimuli, with 22 s intervals), at intensity of 120% RMT. A blank-coded magnetic card, able to activate the real or sham modality on the H-coil controller was randomly assigned to each participant and the reading codes were kept by administrative personnel not involved in the study. This procedure ensured blindness of patients and operators administering rTMS. In addition, the assessing clinicians and neuropsychologists were kept away from the stimulation environment to also ensure their blindness. Treatment consisted in 16 rTMS sessions: 4 weeks of full treatment with 3 sessions/week, followed by 4 weeks with only one weekly maintenance session, coupled with interview to patient and caregiver for side effects.

### Clinical Evaluations

Each patient was clinically evaluated at 4 time-points: at baseline (t0), after 4 weeks of treatment (t1) and after the maintenance treatment period which included 4 additional weekly sessions (t2). A follow-up evaluation at 4 months was also carried out (t3). Subjects underwent a complete neurological examination with side effect reporting and neuropsychological testing including: MMSE (Mini Mental State Examination), BDI-II (Beck Depression Inventory scale-II), CGI-I (Clinical Global Impression-Improvement) and ADAS-cog (Alzheimer's Disease Assessment Scale-cognitive) (37–40). The ADAS-cog consists of 11 cognitive items and its total score ranges from a minimum of 0 (absence of cognitive deficits) to a maximum of 70 (severe cognitive deficit). Similarly to MMSE, ADAS-cog is a global tool for testing several cognitive domains (i.e., memory, language, comprehension, praxis, orientation). However, the MMSE is a screening test with relatively modest sensitivity and high inter-session variability. It has floor and ceiling effects and limited sensitivity to change. This limits its wider use in detecting change in clinical work and in research studies. For this reason ADAS-cog is preferable to assess cognitive benefits induced by an experimental treatment on AD patients (41). The Italian version of the ADAS-cog was used (M. Fioravanti, Giunti Organizzazioni Speciali, Firenze). Since lower ADAS-cog raw scores indicate better cognitive function, a negative post-treatment change from baseline represents a clinical improvement. BDI-II improvement was considered as a reduction from baseline values, as well; on the contrary, improvement in MMSE score is reflected by a positive change at follow up. Finally for CGI-I, which is a 7-point scale ranging from 1 (very much improved) to 7 (very much worse), the lower the score the higher the improvement from baseline is. Vital signs (blood pressure and heart rate) were recorded before and after each rTMS session as an additional safety measure.

### Statistical Analyses

The primary analyses were conducted using a mixed effects linear model (random coefficients model) which adjusts for missing data in testing for differences in the intercepts (baseline scores) and slopes (rate of change) of the ADAS-Cog, MMSE, CGI-I, and BDI between treatment groups. For each of the outcomes, a model was constructed with treatment effect, time effect, and treatment x time interaction term, with age, sex, years of education, disease duration as covariates. Each model included random effects of time at the individual level after comparing the log-likelihoods of models with and without random effect. An unstructured covariance matrix was used to model the independence of the slope and intercept parameters. Parameters were estimated using restricted maximum likelihood. The primary test of interest was the significance of the treatment by time interaction. To investigate the magnitude of rTMS efficacy, group differences have been tested from t0 to t2, while to assess whether effects lasted beyond the last rTMS session we have included t3 as well. Group differences in normally distributed variables, shown as mean (SD), were analyzed using unpaired *t*-tests, while those in non-normally distributed variables, shown as medians with 25 and 75% percentiles, were explored with the Mann-Whitney *U*-test. Differences in categorical variables, shown as proportions, were analyzed using χ^2^-tests. A two-sided *p*-value of 0.05 was considered statistically significant. All statistical analyses were performed using the computing environment R (42).

## Results

A total of 28 subjects (mean age 70.9 years, SD 8.1, female:male ratio 1:1) were included in the analyses. Sixteen patients have been treated with real rTMS, while twelve have been treated with sham rTMS (Table 1). Resting Motor Threshold (RMT) did not significantly differ between “sham” and “real” groups, neither at baseline (*p* = 0.59) nor at t1 (*p* = 0.27) and t2 (*p* = 0.41).

**Table 1:** Demographics and neuropsychological scores at baseline.

**Characteristics**	**Levels**	**All patients**	**Sham rTMS**	**Real rTMS**
Sex	N° Females (%)	13 (46.4)	6 (50.0)	7 (43.8)
	N° Males (%)	15 (53.6)	6 (50.0)	9 (56.2)
Age	Mean years (SD)	70.9 (8.1)	72.6 (8.3)	69.6 (7.9)
Education	Mean years (SD)	8.6 (4.1)	7.8 (3.4)	9.2 (4.5)
Disease duration	Mean years (SD)	4.2 (1.7)	4.2 (1.1)	4.2 (2.0)
ADAS-cog total score	Mean (SD)	32.0 (13.9)	35.0 (15.8)	29.7 (11.9)
MMSE	Mean (SD)	16.9 (5.5)	15.5 (5.6)	18.0 (5.3)
BDI	Mean (SD)	6.1 (6.1)	6.4 (6.6)	5.9 (5.9)
CGI	Mean (SD)	4 (0.4)	4 (0.4)	4 (0.4)

At baseline, there were no significant group differences in age, sex, education, disease duration and neuropsychological profile (ADAS-cog, MMSE, CGI-I, and BDI-II).

**Figure 1:**
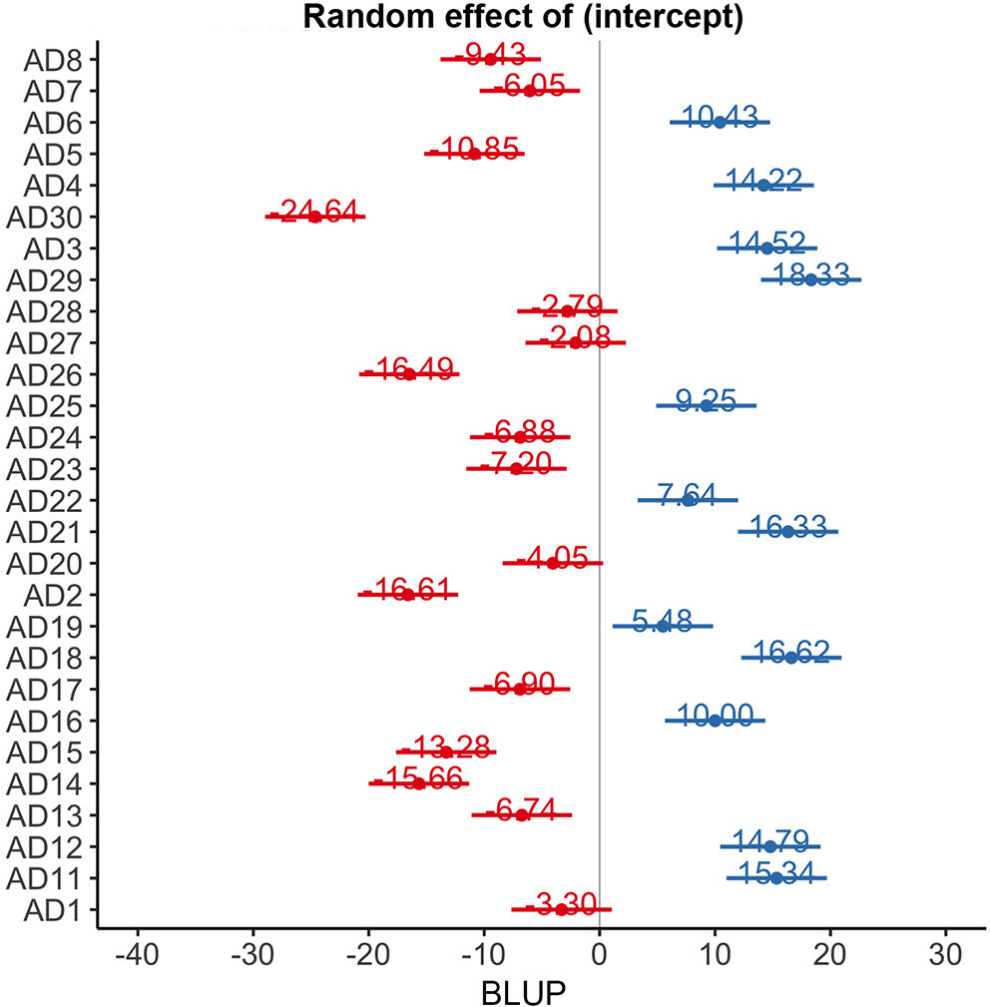
Random effect of time on ADAS-cog scores at an individual level. BLUP, best linear unbiased predictor.

**Figure 2:**
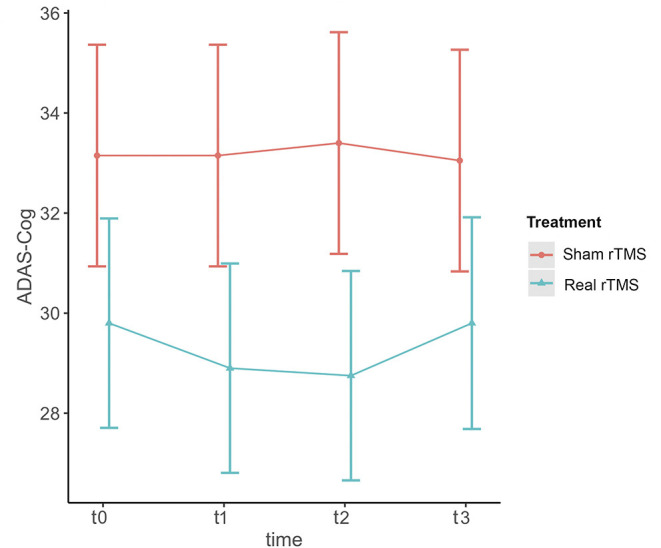
Effect of rTMS on ADAS-cog scores over the treatment period and the observational follow-up. T0 = baseline; T1 = 1 month after randomization; T2 = 2 months after randomization corresponding to the end of treatment; T3 = 4 months after randomization, corresponding to two months after the end of treatment. The figure represents predicted means ± standard errors.

ADAS-Cog scores changes over time varied significantly at an individual level (Figure 1). A trend toward lower ADAS-cog scores at the end of treatment was observed for patients treated with real rTMS in comparison to sham-treated- patients. Specifically, rTMS-treated patients had a mean decrease in ADAS-cog score of −1.01 (95% CIs −0.02 to −3.13, *p* < 0.04) per time point (Figure 2 and Table 2) in comparison to sham-treated patients. This difference disappeared at t3, after 2 months the treatment was over (Figure 2). Real rTMS, in comparison to sham rTMS, showed no effects on MMSE, CGI-I, and BDI changes over time.

**Table 2:** Mixed effects (random coefficients) model of ADAS-Cog change over time.

	**B**	**95% CIs**		***p***
Age—per year increase	−0.06	−0.81	0.69	0.87
Sex—Male vs. female	−3.45	−15.71	8.81	0.57
Education—per unit increase	−0.42	−2.27	1.42	0.64
Disease duration—per unit increase	0.14	−3.29	3.58	0.93
Time—months	−0.28	−1.72	1.17	0.70
Treatment	−4.25	−16.20	7.69	0.47
Time × treatment	−1.01	−0.02	−3.13	0.04

Concerning the safety of rTMS, none of the enrolled subjects reported serious side effects related to treatment. A patient belonging to the sham group had an acute myocardial infarction after 2 weeks of treatment, away from the rTMS sessions, considered unrelated to the participation in the study. After one rTMS session, the same patient reported an episode of transient headache. The procedure was overall well-tolerated, with the exception of one patient of the real group, who did not tolerate the intensity of 120% RMT in 11 out of 16 rTMS sessions, requiring lowering it at 95–110% RMT.

## Discussion

To our knowledge, this is one of the first studies to have tested feasibility, safety, and efficacy of bilateral simultaneous rTMS with H-coil as add-on symptomatic treatment in AD. The design of the present study is based on prior open-label experience (32) in which AD patients reported improvement in cognitive functions after rTMS with H2-coil using the same parameters of the present study. We aimed at confirming these preliminary results in a prospective double-blind study.

As this was a pilot study, we aimed at measuring the specific contribution to a possible cognitive improvement of r-TMS with H-coil alone, without any combined cognitive training. Nevertheless, growing evidence suggests that the on-line approach, i.e., the combination of r-TMS and cognitive tasks during the stimulation sessions, offer the best results (8, 43). Our study demonstrates significant, even though transient, beneficial effect of rTMS on global cognition as measured with ADAS-COG. We did not find any significant improvement in MMSE score, instead. Even if MMSE is a global neuropsychological tool to test for patients' cognition, such as ADAS-cog, this is rather predictable since MMSE is a very generic screening tool with floor and ceiling effects and scarce sensitivity to detect clinical improvements (41).

The limited rTMS effect in our sample is not entirely surprising, considering the lack of associated cognitive training and the relatively advanced disease stage of some patients included. In fact, as shown in a previous study (8), the concept of “brain reserve” is critical not only for determining the expression of cognitive decline in the natural evolution of the disease (44–46), but also for the impact of possible treatments. Our sample had a relatively severe cognitive impairment, and lack of cortical plasticity to rTMS has been found associated with a more severe rate of cognitive decline (47) and to predict less benefits from rTMS treatment associated with cognitive rehabilitation (11). We can therefore argue that rTMS may be potentially more effective especially when applied in the earlier disease stages, when the neurodegenerative process is not overwhelming and there are still preserved neuronal networks to be stimulated and strengthened by rTMS.

On the other hand, the benefits of non-invasive brain stimulation on higher brain function in the elderly and in AD are greater when cognitive training is combined, as evidenced in a meta-analysis by Hsu (43). It is widely accepted that rTMS acts on brain, at least in part, by enhancing synaptic plasticity through LTP (48–50). Since synchronous stimulation of two neurons results in synaptic LTP, a long lasting enhancement in inter-neuronal signal transmission and a crucial element of synaptic plasticity, we think that a simultaneous stimulation of neuronal circuits by both r-TMS and cognitive training may enhance or consolidate the effects obtained with one single tool, as already emerged from previous evidence on AD subjects (8, 51). In this context, it is still unknown which type of stimulation pattern and distribution would best facilitate the enhancement of LTP mechanisms. While we may hypothesize that a focal stimulation could better suit the circuit-specific nature of LTP in crucial hubs, it is also possible to speculate that the simultaneous stimulation of several relevant regions within the same network could have the advantage of acting through cortico-cortical connections. Furthermore, it is also possible that when brain stimulation alone is applied—independently from the coil type—further mechanisms might be involved. At least two other mechanisms may be speculated: rTMS may increase Brain Derived Neurotrophic Factor (BDNF) levels and enhance N-methyl-D-aspartate receptor (NMDAR) expression in cortical neurons, as demonstrated in an animal model of vascular dementia (52). An increase in plasma BDNF levels after rTMS was confirmed by studies on human subjects suffering from other neurodegenerative disorders, such as Amyotrophic Lateral Sclerosis (53, 54). Brain Derived Neurotrophic Factor is one of the most important neurotrophic factors, involved in several neuronal protective mechanisms against injury (55). It is not known whether the wider stimulation region of the H-coil may further enhance this mechanism compared with focal stimulation or the opposite. On the other hand, postsynaptic activation of NMDARs induce Ca^2+^ influx which triggers a series of reactions that lead to long-term changes in synaptic strength (56). Therefore, given the lack of a synergic effect guaranteed by simultaneous cognitive training, it is not surprising that in the present study the advantage of active treatment over sham was transient. This finding is in partial disagreement with other r-TMS studies that demonstrated a persistent cognitive improvement outlasting the end of treatment in real r-TMS groups. Even if a direct comparison between the present and other studies could be rather difficult due to differences in the sample characteristics, treatment sessions, type of coil adopted and number of stimuli, outcome variables used or association with cognitive training, nevertheless some considerations can be argued. Up to now, few longitudinal study that used ADAS-cog as outcome variable. Among those, Rabey et al. (8) used a figure-of-eight coil to apply rTMS over six different cortical regions, primarily involved in the manifestation of AD symptoms, in combination with a cognitive training specific to the stimulated area. The protocol consisted of an intensive phase of 5 stimulation days a week for 6 weeks, followed by a biweekly maintenance for 3 months. ADAS-cog significantly improved in the active compared with sham treatment and the effect persisted at follow-up, differently from the present study. In our opinion this not negligible difference can be due to several reasons: the longer duration of treatment period and maintenance phase, the higher number and frequency of rTMS sessions, the association with a targeted cognitive training. In particular we may hypothesize that performing cognitive tasks specific to cortical regions typically involved in AD simultaneously with r-TMS sessions on the same areas could have determined a synergic effect on synaptic LTP, thus guaranteeing a long lasting cognitive improvement in the real group. Nevertheless, improvement in certain cognitive domains persisting at follow-up was also reported in two studies using rTMS without cognitive training (3, 4). Cotelli et al. (4) reported significant improvement in sentence comprehension, but not in other cognitive domains, 8 weeks after the end of a 4 week-rTMS treatment applied over the left DLPFC in 5 AD patients, compared to 5 patients who received 2 weeks of sham rTMS followed by 2 weeks of real rTMS. In the present study we did not use specific neuropsychological tools to test for sentence comprehension, but the lack of cognitive improvement in other cognitive tasks is consistent with our findings. In the study by Ahmed et al. (3), cognitive improvement was present only in mild to moderate and not in severe AD patients and persisted after 3 months from the end of treatment. That finding supports the view that r-TMS is more effective when applied in earlier rather than in later AD phases and is in line with our finding that patients less impaired at baseline tended to show greater improvement after 1 month real rTMS. However, we may consider that scores on MMSE were higher in the 20 Hz group even before the application of rTMS in that study.

Another possible factor accounting for differences in magnitude and duration of rTMS effects between our study and previous studies could be the number and spacing of the weekly rTMS sessions (e.g., daily as in the previous studies vs. interspaced as in the present study), besides association with specific cognitive tasks. Finally, our number of stimuli (840/session) was considerably lower compared with previous studies using multi-site stimulation (1,300/session) associated with cognitive training [e.g., (8)].

In the present study we did not use neurophysiological markers to probe the effects of rTMS, such as EEG-derived measures like event-related potentials, so we could not objectively measure possible functional changes in relevant cognitive pathways such as pre-frontal dopaminergic networks and parietal norepinephrine-dependent pathways. Another limitation of our study is the possible underestimation of the “placebo effect” in patients receiving sham stimulation: even though sham stimulation revealed to produce the same physical and psychological sensations as the real one (36), in future studies it would be useful to ask the patients to guess whether they received active or sham stimulation.

## Conclusions

Based on these considerations, our preliminary findings suggest that rTMS with the H-coil is a feasible and safe procedure in AD, with potential benefits on cognition. If confirmed, these results may offer the possibility to provide a new add-on non-pharmacological intervention in AD. The possible advantage over other focal rTMS techniques (e.g., using the figure of eight coil) owing to the simultaneous stimulation of wider neuronal networks and different cortical areas during the same session still needs to be explored, as well as the optimal dose, number of sessions and predictors of treatment response. Future studies on AD should mainly focus on the early disease stages, in order to exploit a higher neuronal and network reserve, and should explore the potential advantage of combination with cognitive training.

## Data Availability Statement

The raw data supporting the conclusions of this article will be made available by the authors, without undue reservation.

## Ethics Statement

The studies involving human participants were reviewed and approved by San Raffaele Hospital Ethical Committee. The patients/participants provided their written informed consent to participate in this study.

## Author Contributions

LL conceived and designed the study, supervised data collection and statistical analyses, performed literature search, data interpretation and manuscript writing and revision. GDC and MP performed statistical analysis, data interpretation, writing and revision of the manuscript. EC and LF performed patient selection, clinical assessments, data collection, literature search, discussion of findings and manuscript revision. RS, MPB, and MF participated in data collection, discussion of findings and manuscript revision. AZ, GM, and GC participated in study conception, design, data interpretation and revised the manuscript for intellectual content.

## Conflict of Interest

AZ was a key inventor of H-coli and acts as a consultant for Brainsway Ltd. The remaining authors declare that the research was conducted in the absence of any commercial or financial relationships that could be construed as a potential conflict of interest.
