# Supplementary material for: Repetitive Transcranial Magnetic Stimulation With H-Coil in Alzheimer's Disease: A Double-Blind, Placebo-Controlled Pilot Study
Source: Front Neurol. 2021 Feb 18;11:614351. doi: 10.3389/fneur.2020.614351 (PMC7930223; doi:10.3389/fneur.2020.614351)
Supplement: Supplementary file 1 [file Data_Sheet_1.docx]

Supplementary material

# Figure 1. Figure 1. Changes of ADAS-cog over time according to treatment arm


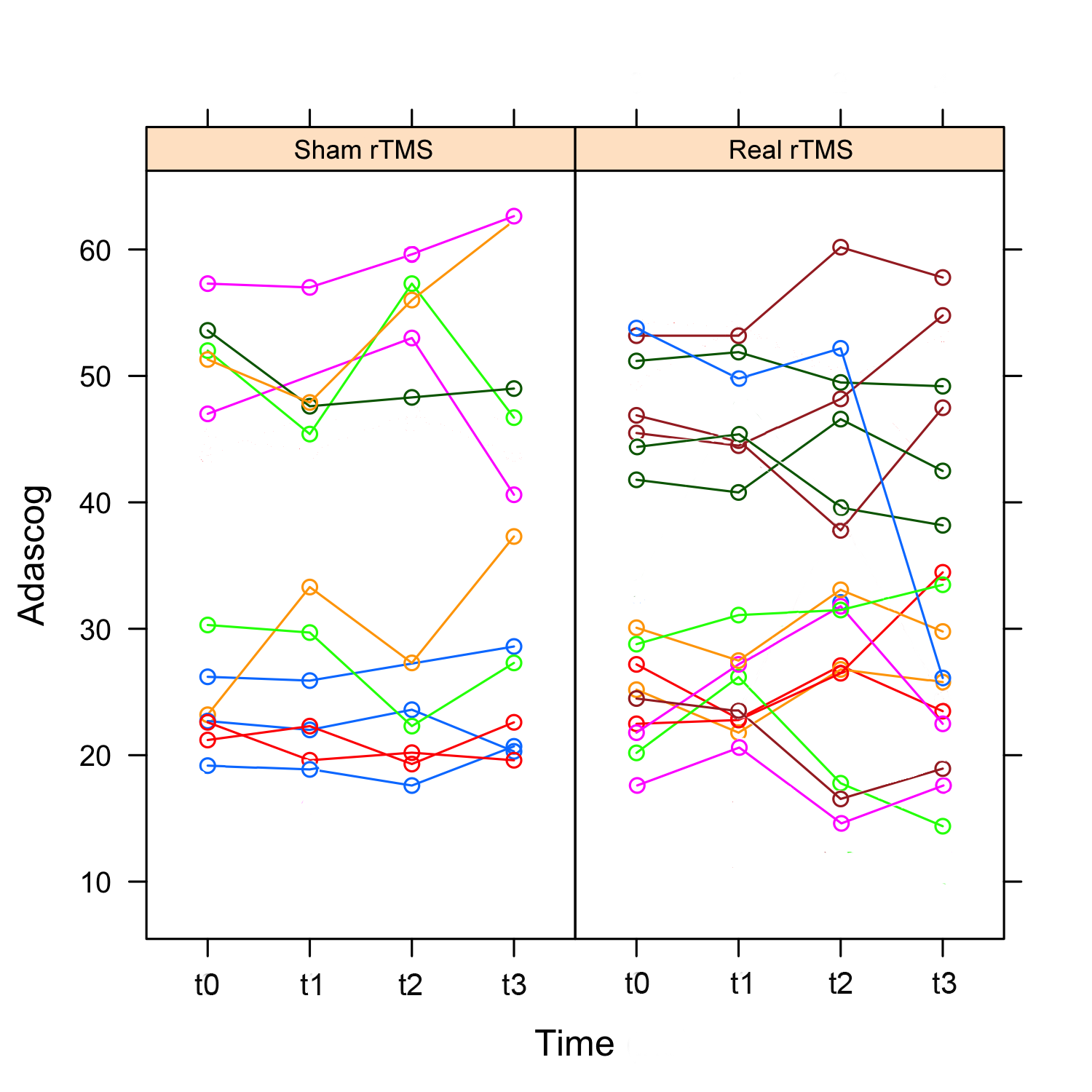


T0=baseline; T1=1 month after randomization; T2=2 months after randomization corresponding to the end of treatment; T3=4 months after randomization, corresponding to two months after the end of treatment
